# Integrating Full-Length Transcriptome and RNA Sequencing of Siberian Wildrye (*Elymus sibiricus*) to Reveal Molecular Mechanisms in Response to Drought Stress

**DOI:** 10.3390/plants12142719

**Published:** 2023-07-21

**Authors:** Qingqing Yu, Yi Xiong, Xiaoli Su, Yanli Xiong, Zhixiao Dong, Junming Zhao, Xin Shu, Shiqie Bai, Xiong Lei, Lijun Yan, Xiao Ma

**Affiliations:** 1 College of Grassland Science and Technology, Sichuan Agricultural University, Chengdu 611130, China; 2 Sichuan Academy of Grassland Science, Chengdu 610097, China

**Keywords:** *Elymus sibiricus*, endemic forage, comparative transcriptomics, drought stress, WGCNA

## Abstract

Drought is one of the most significant limiting factors affecting plant growth and development on the Qinghai–Tibet Plateau (QTP). Mining the drought-tolerant genes of the endemic perennial grass of the QTP, Siberian wildrye (*Elymus sibiricus*), is of great significance to creating new drought-resistant varieties which can be used in the development of grassland livestock and restoring natural grassland projects in the QTP. To investigate the transcriptomic responsiveness of *E. sibiricus* to drought stress, PEG-induced short- and long-term drought stress was applied to two Siberian wildrye genotypes (drought-tolerant and drought-sensitive accessions), followed by third- and second-generation transcriptome sequencing analysis. A total of 40,708 isoforms were detected, of which 10,659 differentially expressed genes (DEGs) were common to both genotypes. There were 2107 and 2498 unique DEGs in the drought-tolerant and drought-sensitive genotypes, respectively. Additionally, 2798 and 1850 DEGs were identified in the drought-tolerant genotype only under short- and long-term conditions, respectively. DEGs numbering 1641 and 1330 were identified in the drought-sensitive genotype only under short- and long-term conditions, respectively. Kyoto Encyclopedia of Genes and Genomes (KEGG) enrichment analysis revealed that all the DEGs responding to drought stress in *E. sibiricus* were mainly associated with the mitogen-activated protein kinase (MAKP) signaling pathway, plant hormone signal transduction, the linoleic acid metabolism pathway, the ribosome pathway, and plant circadian rhythms. In addition, *Nitrate transporter 1/Peptide transporter family protein 3.1* (*NPF3.1*) and *Auxin/Indole-3-Acetic Acid (Aux/IAA) family protein 31*(*IAA31*) also played an important role in helping *E. sibiricus* resist drought. This study used transcriptomics to investigate how *E. sibiricus* responds to drought stress, and may provide genetic resources and references for research into the molecular mechanisms of drought resistance in native perennial grasses and for breeding drought-tolerant varieties.

## 1. Introduction

Drought is a widespread climatic phenomenon that has a profound effect on how plants grow and develop and can significantly affect agricultural production and ecosystems [1]. As the third pole of the earth, the drought-stricken areas of the Qinghai–Tibet Plateau (QTP) account for 63% of the total area of agricultural and grazing land [2], and this problem will likely worsen with global climate change and human activities such as mining that weaken the plateau ecosystem and erode the ecological safety barrier functions of the QTP [3]. The development of germplasm adapted to arid regions on the QTP is essential for the preservation of the ecological functions of this area and for the development of stable livestock production. One of the effective ways is to understand the drought resistance molecular mechanism and improve the drought resistance of the endemic grass species, using biotechnology techniques.

*Elymus sibiricus* (StStHH, 2n = 4x = 28), also known as Siberian wildrye, is considered the model species of the genus *Elymus* in the tribe Triticeae [4]. With its high biomass yield, ease of cultivation, high crude protein content, and good palatability, *E. sibiricus* is a widely distributed perennial grass species in the arid and semiarid temperate regions of Eurasia. It plays a key role as a dominant or constructive species in alpine meadow–steppe-type pastures, particularly in the QTP region [5], due to its strong adaptability to various high-altitude environments, such as its drought tolerance and cold resistance. *E. sibiricus* is commonly used as the primary grass species for constructing artificial grasslands and restoring degraded grasslands in the QTP, and has been instrumental in addressing ecological and livestock development problems in this region [6]. Moreover, *E. sibiricus* is one of the few species that can achieve large-scale local grass seed production in China, and it occupies an important position in the grass seed industry. Therefore, it is of great importance for developing livestock and the maintenance of the ecological function of the QTP to investigate the molecular mechanism of drought resistance, to excavate the drought resistance genes, and to screen or create strong drought-tolerant accessions of *E. sibiricus*.

By triggering a variety of molecular, biochemical, and physiological responses, plants can activate different signaling pathways in response to drought stress [7]. Drought stress causes wilting when water loss exceeds water uptake, often measured by leaf water content (LWC) [8,9,10]. A favorable LWC contributes to the smooth progress of plant physiological and biochemical reactions and various signal pathways in response to drought stress [11]. Researchers have identified potential genes and mechanisms of drought response in different plants by analyzing the enrichment of genes involved in metabolic pathways under drought stress [12]. Understanding how general and specific genes are expressed and regulated in drought-stressed plants is a crucial step [13], which can provide a better understanding of the mechanisms underlying drought tolerance and aid in developing new strategies for improving crop productivity and resilience under water-limited conditions. Drought is a complex natural phenomenon, and the drought stress suffered by plants in the natural environment is often uncertain [14]. Sometimes, plants experience short-term drought stress, where they mainly respond by immediate adjustments like regulating water and ion channels [15]. However, under long-term drought stress, more complex molecular mechanisms may be involved: genes related to photosynthesis may be down-regulated, while genes related to proline metabolism are up-regulated, protein synthesis, DNA methylation, and chromatin remodeling [15,16,17]. Therefore, studying the gene expression patterns of plants under both long-term and short-term drought helps us to better understand how plants respond to drought stress, morphologically, physiologically, and molecularly, enabling them to escape drought or cope with lower water potential [18]. Phytohormone abscisic acid (ABA), one of the best-defined responses to drought and other types of abiotic stress, initiates the transcriptional reorganization that leads to multiple outcomes, including the enrichment of osmoprotectants and the closure of stomata [19]. In addition, various transcription factors (TFs) such as NAC, WRKY, C2H2, MYB, etc. play important roles in the regulation of the expression of multiple genes in plants in responding to the stresses of drought [20,21].

In the present study, the transcriptional responses of two genotypes of wild *E. sibiricus* collections to drought stress were investigated using both the third-generation full-length and second-generation transcriptomes. The aims of this study were: (1) to identify the drought-responsive genes of *E. sibiricus*; (2) to compare the transcriptional responses of two genotypes of *E. sibiricus* to long- and short-term drought stress; (3) between the two very different genotypes to compare the expression patterns of key metabolic pathways and transcription factors in response to drought. This study could provide genetic resources and clues for research into drought resistance in this and other closely related grass species.

## 2. Results

### 2.1. The General Features of the Third-Generation and Second-Generation RNA-Seq

In order to accurately obtain the transcriptomic information of *E. sibiricus*, full-length transcripts were extracted from the mixed samples using Pacbio’s Sequel platform. A total of 45 GB of raw data was acquired, with an average read length of 1727.33 bp, and an average N50 of 1898.33 bp (Table S1), by the third-generation transcriptome sequencing. We obtained 43,620,290.3 circular consensus sequences (CCS), and their average length was 2020 bp (Table S1). A total of 51,215 high-quality sequences and 607 low-quality sequences were identified by hierarchical clustering using similar full-length non-chimeric (FLNC) reads (Table S2). Additionally, 40,708 reliable isoforms were obtained by correcting the full-length sequence and removing redundancy analysis based on Illumina sequencing data (Table S3).

**Figure 1:**
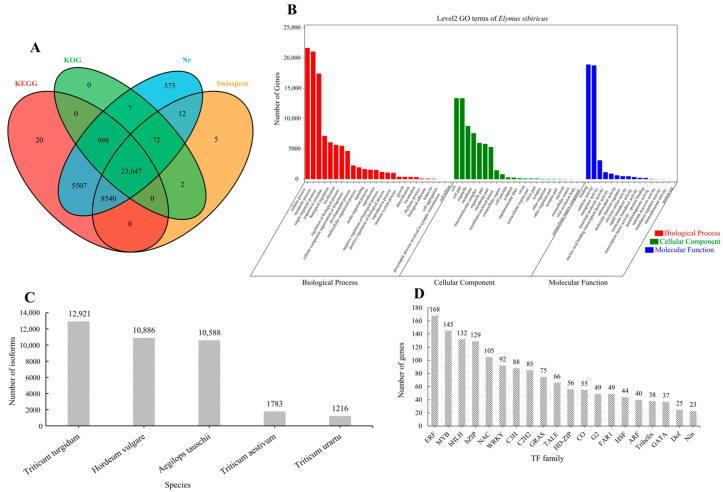
The Venn diagram of the isoform numbers annotated to four databases (**A**). GO annotation results of all isoforms (**B**). Distribution of isoforms annotated in Nr database in different species (**C**). Distribution of transcription factor families predicted by all isoforms (**D**). The data constructed by the figure (**A**–**D**) is based on the three-generation full-length transcriptome data.

The 40,708 high-quality isoforms were searched in the SwissProt, KEGG, Nr, and KOG databases; 96.75% (39,385/40,708) of the isoforms’ annotation was successful (Figure 1A). *Triticum turgidum* (12,921), *Hordeum vulgare* (10,886), *Aegilops tauschii* (10,588), *Triticum aestivum* (1783), and *Triticum urartu* (1216) were the five species with the highest number of isoforms according to the Nr database (Figure 1C). The processes that isoforms were involved in included the cellular process, metabolic process, and single-organism process, whereas cellular component involvement mainly included cells and cell parts. Moreover, the isoforms were involved in molecular functions, mainly including binding and catalytic activity (Figure 1B). We further predicted 1777 isoforms as transcription factors (TFs) belonging to 52 families. Notably, ERF (168), MYB (145), bHLH (132), bZIP (129), NAC (105), and WRKY (92) were identified as drought-stress-responsive TFs in *E. sibiricus* (Figure 1D).

A total of 259,407,116,765 bp of clean data and 1,734,229,420 clean reads were detected using Illumina RNA-seq. Moreover, the Q30 of the clean data ranged from 92.70% to 94.56%, and the GC content ranged from 53.21% to 55.32% (Table S4). The result indicated that the sequencing quality was sufficient for downstream analysis.

### 2.2. Differentially Expressed Genes under Drought Stress

**Figure 2:**
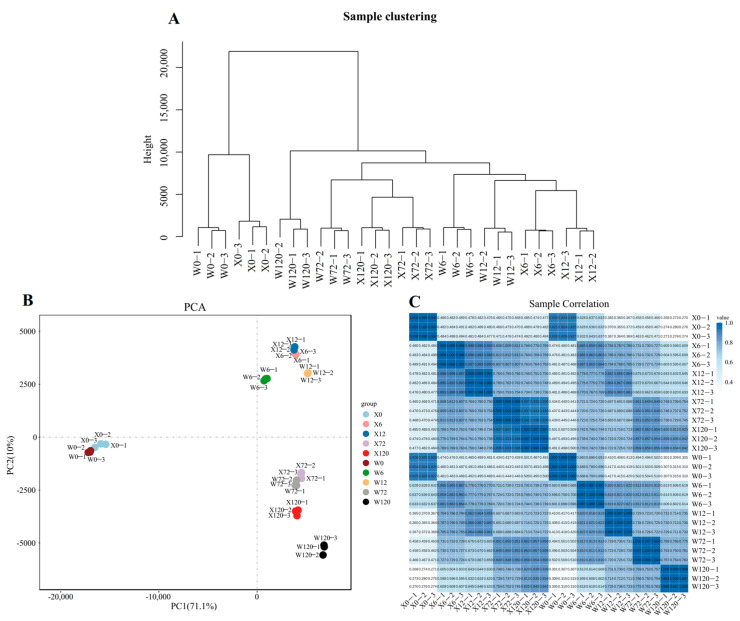
Quality control analysis of second-generation transcriptome data. The hierarchy of 30 samples (**A**). The 2D map of PCA (**B**). The heatmap of Pearson’s correlation of 30 samples (**C**).

PEG6000 was used to simulate drought stress for two genotypes (X and W) and analyzed at different time points (0 h, 6 h, 12 h, 72 h, and 120 h) to study the response of *E. sibiricus* to drought stress. A total of 40,708 isoforms were expressed in 30 samples and the hierarchical cluster analysis confirmed the grouping of three biological replicates under each treatment (Figure 2A). Similarly, the results of principal component analysis based on the all-genes expression of 30 samples demonstrated that the PC1 could explain 71.1% of the total variation, allowing for the successful distinguishment of the CK and treatment group (X0 and W0 vs. drought-stressed samples, Figure 2B). Furthermore, the PC2 explained only 10% of the total variation, but it was able to distinguish between short-term (6 h and 12 h) and long-term (72 h and 120 h) stress, indicating that the *E. sibiricus* showed different gene expression patterns under short- and long-term drought stress (Figure 2B). Pearson’s correlation analysis showed a positive relationship between the three biological replicates (Figure 2C), which was further confirmed by hierarchical clustering and PCA analysis. In addition, the results of the qRT-PCR analysis were basically in agreement with those of the RNA-seq analysis, which further confirmed the reliability of the RNA-seq data (Figure S1).

**Figure 3:**
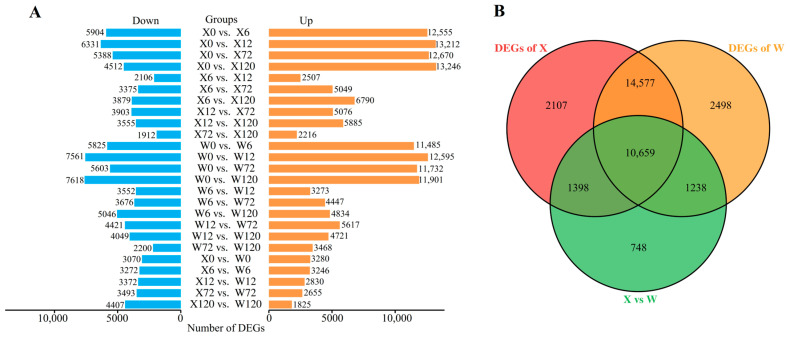
Histogram of DEGs in 25 comparison groups (**A**); Venn diagram of DEGs (**B**), X represents drought-tolerant genotype and W represents drought-sensitive genotype.

Only DEGs exhibiting |log_2_FC| ≥ 1.0 and FDR < 0.05 were considered for subsequent analysis. A total of 33,225 DEGs were detected in 25 comparison groups under drought stress in the two genotypes (Figure 3B). Among these, 10,659 DEGs were common in both two genotypes, whereas 2107 and 2498 DEGs were uniquely identified in the X and W genotypes, respectively. Notably, the number of up-regulated DEGs was substantially higher than the number of down-regulated DEGs in both genotypes under drought stress (Figure 3A).

### 2.3. Gene Expression Patterns between Two Genotypes with Distinct Drought Tolerance

**Figure 4:**
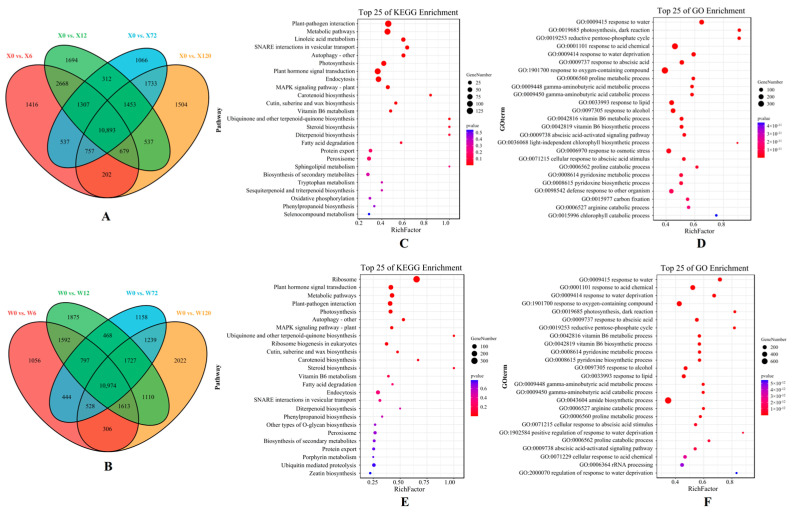
Venn diagram that shows the differentially expressed genes (DEGs) identified in four comparison groups of the X genotype (**A**) and W genotype (**B**). Additionally, KEGG enrichment pathway maps were generated to display the DEGs shared by the four comparison groups of the X genotype (**C**) and W genotype (**E**), while GO enrichment pathway maps were used to illustrate the DEGs shared by the four comparison groups of the X genotype (**D**) and W genotype (**F**). X represents drought-tolerant genotype and W represents drought-sensitive genotype.

The volcano maps based on all the DEGs of two genotypes under different treatments compared to the CK showed that more up-regulated DEGs than down-regulated DEGs were present (Figure S2). Moreover, the number of DEGs up-regulated by the X genotype was greater than that of the W genotype at four treatment time points, confirming that *E. sibiricus* up-regulated more gene expression to increase its drought resistance. In addition, there are 10,893 common DEGs in the four comparison groups (X0 vs. X6, X0 vs. X12, X0 vs. X72 and X0 vs. X120) in the X genotype and 10,974 common DEGs in the W genotype in the four comparison groups (W0 vs. W6, W0 vs. W12, W0 vs. W72 and W0 vs. W120), indicating that there were similar results between these two genotypes (Figure 4A,B).

KEGG pathway and GO enrichment of common DEGs were used to further investigate the differences in drought resistance mechanisms between the two genotypes. In total, 62 and 70 GO terms were enriched in the X and W genotypes, respectively, including response to water (GO: 0009415), response to acid compounds (GO: 0001101), response to water shortage (GO: 0009414), and photosynthesis dark response (GO: 0019685) (Figure 4D,F). Furthermore, the reductive pentose phosphate cycle (GO: 0019253), the basic cycle of carbon dioxide fixation in C3 plants, was uniquely enriched in the top five GO terms in the X genotype. It is noteworthy that one KEGG pathway (SNARE interactions in vesicular transport, a key metabolic pathway for transmembrane transport in the cytoplasm) was uniquely and significantly enriched in the X genotype.

### 2.4. Responses of E. sibiricus under Short-Term and Long-Term Drought Stress

Venn plots of DEGs for different drought periods were constructed to explore the differential gene regulation in two different genotypes under short- and long-term stress. It was found that 1484 and 1314 DEGs of the X genotype were up- and down-regulated only under short-term drought stress, and 1194 and 656 genes were up- and down-regulated only under long-term drought stress (Figure S3). Similarly, 958 and 683 DEGs were uniquely up- and down-regulated under short-term drought stress in the W genotype, and 739 and 591 genes were uniquely up- and down-regulated under long-term drought stress (Figure S3).

**Figure 5:**
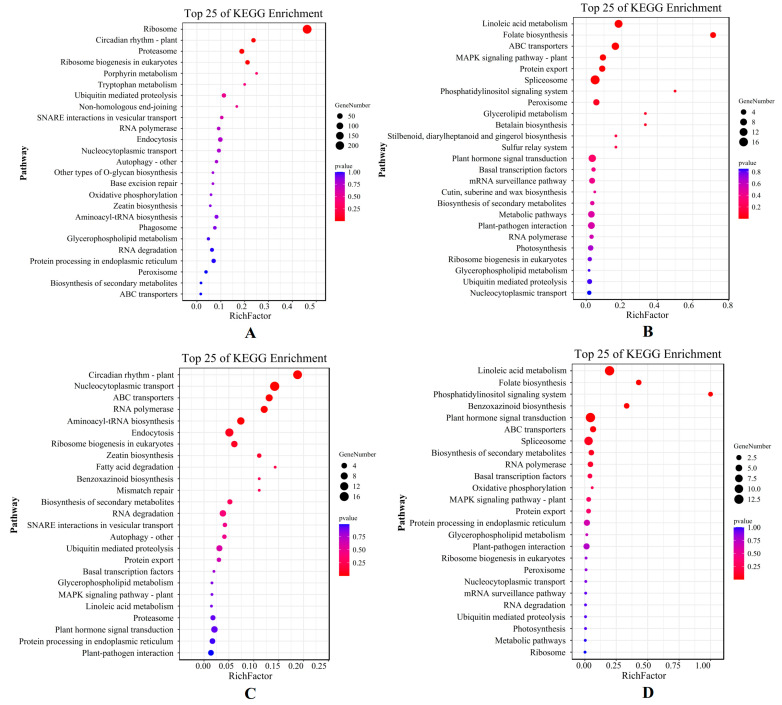
KEGG enrichment pathway map of DEGs by X genotype only under short-term (**A**) and long-term (**B**) drought stress; KEGG enrichment pathway map of DEGs by W genotype only under short-term (**C**) and long-term (**D**) drought stress. X represents drought-tolerant genotype and W represents drought-sensitive genotype.

The result of the KEGG analysis showed that the top five pathways, which mainly included the ribosomal pathway, the plant circadian rhythm, the proteasome, ribosomal biogenesis in eukaryotes, and the porphyrin metabolism pathway, were enriched in the X genotype only under short-term stress (Figure 5A). On the other hand, linoleic acid metabolism, folate biosynthesis, ABC (ATP-binding cassette) transporter, MAPK signal pathway, and protein export pathway were enriched only under long-term stress (Figure 5B). For the W genotype, the top five KEGG pathways annotated only under short-term stress were the plant circadian rhythm, nucleocytoplasmic transport, the ABC transporter, RNA polymerase, and the aminoacyl-tRNA biosynthesis pathway (Figure 5C), whereas linoleic acid metabolism, folate biosynthesis, the phosphatidylinositol signaling system, benzoxazinoid biosynthesis, and the plant hormone signal transduction pathway were enriched only for long-term stress (Figure 5D). The DEGs of *E. sibiricus* under short-term drought were mainly involved in ribosome enrichment and growth and development, indicating that *E. sibiricus* was less affected by drought in the early stages of drought. In addition to providing energy for transcription and translation, there was also sufficient energy for growth and development. Under long-term drought stress, both genotypes showed similar metabolic processes, mainly through linoleic acid and folate biosynthesis, to resist severe drought stress.

### 2.5. Gene Expression Pattern of E. sibiricus in Response to Drought Stress

**Figure 6:**
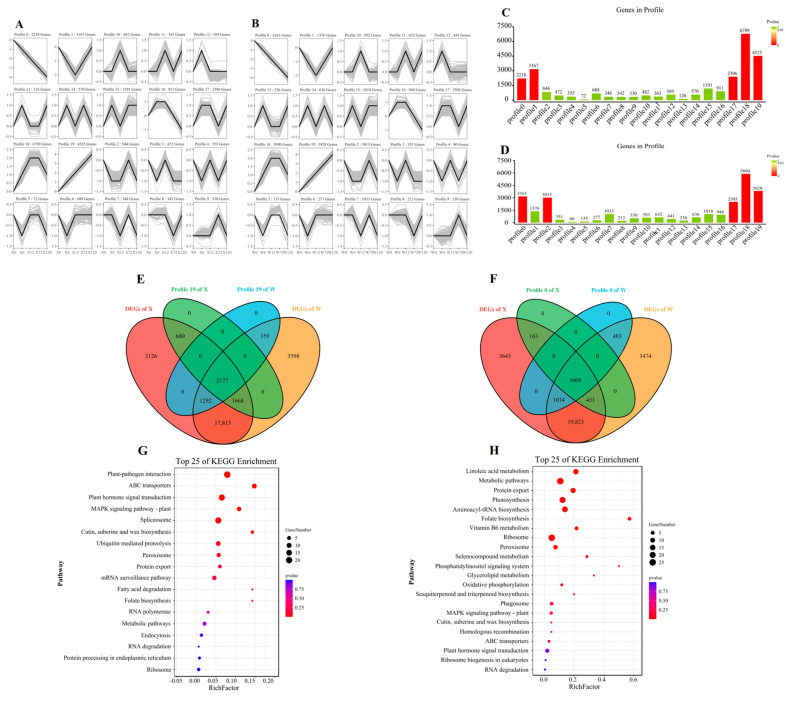
Trend analysis of all genes in X genotype (**A**) and W genotype (**B**). Histogram of the number of genes per profile in X genotype (**C**) and W genotype (**D**). The Venn diagram of DEGs and genes of profile in X genotypes and W genotypes, (**E**) represent profile 19 and (**F**) represent profile 0; KEGG enrichment pathway map of DEGs in profile 19 (**G**) and profile 0 (**H**). X represents drought-tolerant genotype and W represents drought-sensitive genotype.

The trend analysis based on all genes was constructed to explore the gene expression pattern of *E. sibiricus* under drought stress (Figure 6A,B). The analysis revealed a total of 20 profiles. Specifically, profiles 0, 1, 17, 18, and 19 in the X genotype and profiles 0, 2, 17, 18, and 19 in the W genotype were found to be significant. (Figure 6C,D). The 2177 and 1604 DEGs in profile 0 and profile 19 were continuously up-regulated and down-regulated, respectively (Figure 6E,F). The DEGs in profile 0 were mainly involved in signal transduction and protein transport, and were annotated in plant–pathogen interaction, ABC transporters, plant hormone signal transduction, MAPK signaling pathway, and spliceosome (Figure 6G). In contrast, 1604 DEGs in profile 19 were mainly associated with metabolic function and photosynthesis in the process of drought resistance in *E. sibiricus*, and were enriched in linoleic acid metabolism, metabolic pathways, protein export, photosynthesis, and aminoacyl-tRNA biosynthesis (Figure 6H). Our findings suggest that *E. sibiricus* uses different molecular mechanisms to adapt to drought stress, with signal transduction and protein transport being critical for drought tolerance in the resistant genotype, whereas metabolic function and photosynthesis play a crucial role in the W genotype. 

### 2.6. Key Drought Stress Response Pathways and Genes Identified

**Figure 7:**
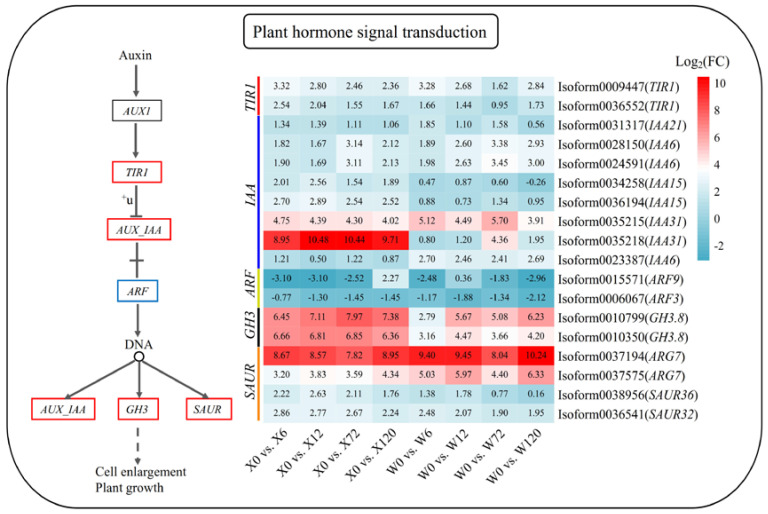
Plant hormone signaling pathway in response to drought stress in *E. sibiricus* based on the KEGG database. The values of the heatmap represent the fold difference between the comparison groups. X represents drought-tolerant genotype and W represents drought-sensitive genotype.

Phytohormone signaling plays a key role in how plants respond to stress. The metabolic pathway map of plant hormone signal transduction in response to drought stress in *E. sibiricus* was constructed based on the published literature and the present analysis results. This pathway identified a total of 18 DEGs. Interestingly, except for indole acetic acid 15 (*IAA15*, *Isoform0034258*), decomposing genes auxin response factor 9 (*AFR9*, *Isoform0015571*) and auxin response factor 3 (*ARF3*, *Isoform0006067*), all DEGs were up-regulated under drought stress compared to the control group (X0 and W0) (Figure 7). In addition, 7 *IAA* genes identified were up-regulated, with *IAA31* (*Isoform0035218*) showing high up-regulation in the X genotype. This suggests that the strong drought tolerance of this genotype may be largely due to the specific up-regulation of *IAA31* (*Isoform0035218*).

**Figure 8:**
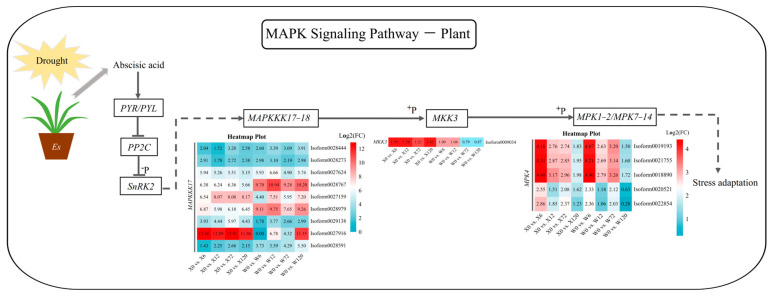
MAPK signaling pathway in response to drought stress in *E. sibiricus* based on the literature and KEGG database. The values of the heatmap represent the fold difference between the comparison groups. X represents drought-tolerant genotype and W represents drought-sensitive genotype.

The MAKP signaling pathway is an essential mechanism for stress adaptation in plants. Abscisic acid (ABA) can improve a plant’s ability to adapt to or resist stress by acting on its downstream genes and regulating the differential expression of related genes when plants are exposed to drought, salt, and osmotic stress. A total of 15 up-regulated DEGs were detected in this pathway for both genotypes, including mitogen-activated protein kinase kinase kinase 17 (*MAPKKK17*), mitogen-activated protein kinase kinase 3 (*MKK3*) and mitogen-activated protein kinase 4 (*MPK4*) (Figure 8). Two *MAPKKK17* genes (*Isoform0028767* and *Isoform0027916*) were highly up-regulated in the W and X genotypes, respectively. Phosphorylated *MAPKKK17* can promote the expression of *MKK3*; however, only one *MKK3* was detected in the MAPK signaling pathway. This *MKK3* was differentially up-regulated at the four drought treatment time points in the X genotype, but only transiently up-regulated under short-term drought in the W genotype, with no significant up-regulation under long-term drought (Log_2_(FC) < 1) (Figure 8). *MKK3*, in turn, promotes *MPK* expression through phosphorylation. Five MPKs were detected in both genotypes, among which two *MPK4* genes (*Isoform0020521* and *Isoform0022854*) were detected in the W genotype under 120 h drought stress. But their differential expression was not significant, possibly due to the lack of differential expression of their upstream *MKK3* under long-term drought stress.

**Figure 9:**
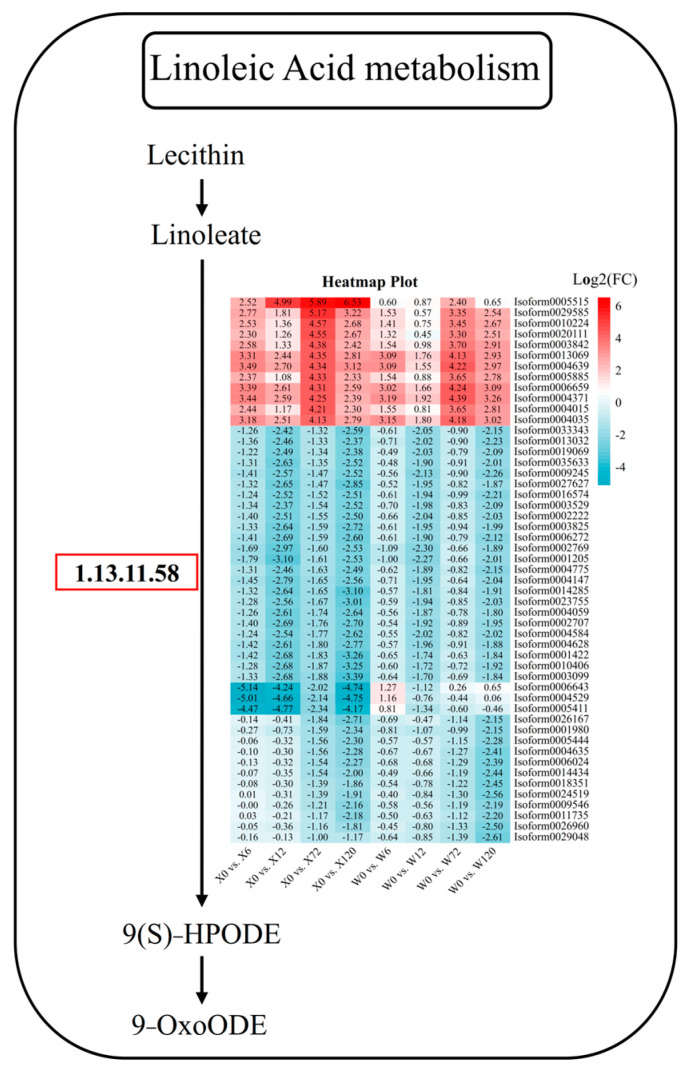
Linoleic acid metabolic pathway in response to drought stress in *E. sibiricus* based on the literature and KEGG database. The values of the heatmap represent the fold difference between the comparison groups. X represents drought-tolerant genotype and W represents drought-sensitive genotype.

In addition to hormones and signaling pathways, some metabolic pathways are also important ways for plants to resist stress, such as flavonoid metabolism, folic acid metabolism, linoleic acid metabolism, and other primary or secondary metabolites. Linoleic acid is a polyunsaturated fatty acid, one of the essential nutrients for the human body, and also one of the important constituents in plants. Linoleic acid fulfills a variety of important physiological functions in plants, including cell membrane composition, signal transduction, and antioxidation. It can increase the fluidity and stability of plant cell membranes, regulate plant growth hormone synthesis and signal transduction, and activate the plant’s antioxidant system to improve the water permeability and drought tolerance of cell membranes. In the linoleic acid pathway, 51 DEGs were detected, among which there were 12 and 39 DEGs up-regulated and down-regulated, respectively (Figure 9). Among the 12 up-regulated genes, *Isoform0005515* was specifically up-regulated in all four treatments of the X genotype, which was considered a key gene in the linoleic acid pathway in responding to stress in *E. sibiricus*. Among the 39 down-regulated genes, three genes (*Isoform0006643*, *Isoform0004529*, and *Isoform0005411*) were down-regulated at four treatment time points in the X genotype, and were considered to be responsible for the difference between the X and W genotypes (Figure 9). Furthermore, other genes, such as *Isoform0026167*, *Isoform0005444*, *Isoform0004635*, *Isoform0006024*, *Isoform0014434*, *Isoform0018351*, *Isoform0024519*, *Isoform00095046*, *Isoform00011735*, *Isoform0026960*, and *Isoform0029048*, showed no difference between short-term drought stress and CK. However, the genes were differentially down-regulated during long-term drought stress in both genotypes and could therefore be considered to be key genes in responding to long-term drought stress in *E. sibiricus* (Figure 9).

### 2.7. WGCNA Identifies Drought Stress Response Transcription Factors and Genes

**Figure 10:**
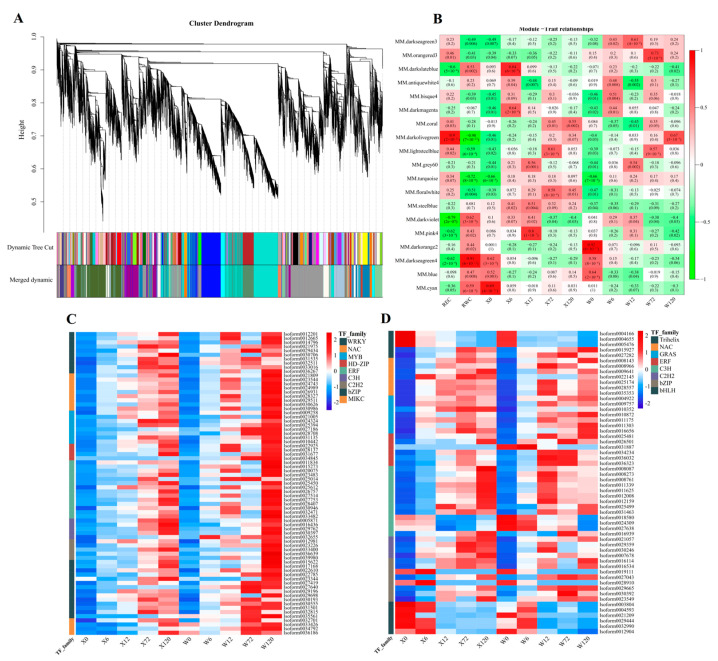
Diagram of the results of WGCNA using 40,708 genes with REC and RWC and 10 samples (**A**); heatmap of Pearson’s correlation of 19 modules with REC and RWC and 10 samples (**B**), the value represents R^2^, and the value in parentheses represents the *p* value. Expression heatmap of differentially expressed transcription factor of darkolivegreen (**C**) and darkseagreen4 (**D**) module in the two genotypes. X represents drought-tolerant genotype and W represents drought-sensitive genotype.

All 40,708 genes were used to construct a weighted gene co-expression network analysis (WGCNA) to explore the relationship between genes in responding to drought stress. A total of 19 modules were identified, each represented by a different color (Figure 10A). Among these, the darkolivegreen module (including 2714 genes) was negatively correlated significantly with RWC, but positively correlated significantly with REC (Figure 10B). On the other hand, the darkseagreen4 module (including 2308 genes) was significantly positively correlated with RWC (Figure 10B).

The 90 and 74 (Figure S4) differentially expressed TFs in the darkolivegreen and darkseagreen4 modules were used to construct expression heatmaps. The major differentially expressed transcription factor families in the darkolivegreen module were WRKY, NAC, MYB, HD-ZIP, ERF, C3H, C2H2, bZIP, and MIKC (Figure 10C), and the expressed levels of these TF families were continuously up-regulated with the duration of drought stress. Additionally, the expression of the W genotypes was higher than that of the X genotypes at 120 h, indicating that the W genotypes were subjected to greater drought stress, and proving that the W genotype is less arid than X genotype. In contrast, the main differentially expressed TF families in the darkseagreen4 module were Trihelix, NAC, GRAS, ERF, C3H, C2H2, bZIP, and bHLH (Figure 10D). Interestingly, one particular TF family, bHLH, showed an opposite expression pattern to other TF families, suggesting that the bHLH family adapts to drought stress by down-regulating expression in *E. sibiricus*.

**Figure 11:**
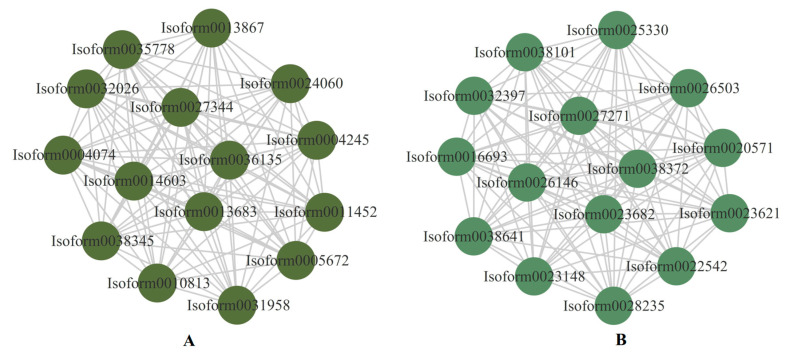
The top 15 hub genes in the proposed modules and relationships within the module. (**A**) represents darkolivegreen and (**B**) represents darkseagreen4.

The top 15 genes in the darkolivegreen and darkseagreen4 modules with correlations greater than 0.8 were used to construct the interaction network graph, and were considered to be the central hub genes (Figure 11). Two genes (*Isoform0027344* and *Isoform0038345*) were involved in the photosynthesis pathway, and the GO function of the two genes was oxidoreductase activity (Table S5). Moreover, four genes (*Isoform0022542*, *Isoform0023148*, *Isoform0026503,* and *Isoform0032397*) were identified as being responsible in photosynthetic organisms for carbon metabolism, and the GO function of the four genes was carbohydrate phosphatase activity (Table S6). One nitrate transporter family (NRT1/PTR, NPF) protein (*Isoform0011452*) was involved in the transmembrane transporter activity (Table S5). These genes are regarded as key genes that have been identified as responsible for drought resistance in *E. sibiricus*. Furthermore, a heatmap of the expressed levels of the 30 hub genes was constructed to assess the pattern of these genes’ regulation of drought resistance in *E. sibiricus*. It was found that the 15 genes in the darkseagreen4 module were down-regulated by drought-induced expression in *E. sibiricus*. Four genes (*Isoform0027344*, *Isoform0032026*, *Isoform0038345,* and *Isoform0035778*) were up-regulated under short-term drought and CK, but down-regulated under long-term drought in both genotypes, and another 11 genes were oppositely expressed in the darkolivegreen module (Figure S5).

## 3. Materials and Methods

### 3.1. Plant Materials, Drought Treatments and Sample Collection

In our previous research, based on the drought resistance evaluation of *E. sibiricus* germplasm resources, drought-tolerant (XJ030-21, X) and drought-sensitive (W16-29, W) accessions were screened [22]. In a growth chamber with a photo period of 16 h of light and 8 h of dark at a temperature of 25 °C, two different genotypes of *E. sibiricus* were grown from seedlings. Clean river sand was used to grow the plants in the pot. To induce stress, two genotypes of *E. sibiricus* were subjected to different durations of treatment with 30% PEG and half-Hoagland’s nutrient solution. The treatments included no exposure (X0 and W0), short-term exposure for 6 h (X6 and W6) and 12 h (X12 and W12), and long-term exposure for 72 h (X72 and W72) and 120 h (X120 and W120). The complete second true leaf of each plant was collected at each treatment time point for both genotypes, with three biological replicates under 5 treatments, resulting in a total of 30 samples. After snap freezing in liquid nitrogen, leaves were stored at −80 °C until extraction.

### 3.2. RNA Extraction and Sequencing

A Trizol kit (Life Technologies, Carlsbad, CA, USA) and its instructions were used to extract total RNA from 30 *E. sibiricus* leaf samples. Concentration and purity-tested qualified RNA were enriched using oligo (DT) magnetic beads. Equal amounts of enriched mRNA from 30 samples were pooled into 3 large mixed samples (10 samples of five treatments of X and W; 1 mixed sample was a biological replicate). To obtain cDNA, the Smarter PCRcDNA Synthesis Kit (Clontech, Mountain View, CA, USA) was used to reverse transcribe mRNA from three mixed samples. The cDNA was then converted to double-stranded DNA using the optimized number of cycles. In further steps, cDNA fragments larger than 5 kb were screened using the BluePippin™ size selection system and equal proportions were mixed with unselected cDNA. The SMRTbell library construction process was then continued by large-scale PCR (Polymerase Chain Reaction). DNA damage repair, terminal repair, and adapter binding were performed on the cDNA. SMRTbell templates were generated using the PacBio Sequel II platform from Gene Denovo Biotechnology Co. (Guangzhou, China) by annealing sequencing primers and polymerase to cDNA.

Qualified RNA from 30 samples was reverse transcribed into cDNA, purified, subjected to end repair and ligated into Illumina sequencing adapters. After size selection by agarose gel electrophoresis, the ligated products were PCR-amplified and sequenced on an Illumina HiSeqTM 4000 platform from Gene Denovo Biotechnology Co. (Guangzhou, China).

### 3.3. Analysis of Full-Length Transcriptome Data

The Isoform Sequencing (IsoSeq) pipeline developed by Pacific Biosciences [23] was employed to analyze the raw sequencing reads obtained from the library. Firstly, from the BAM files of the sub-reads, high quality circular consensus sequences (CCS), also referred to as HiFi reads, were generated. If all of the CCS reads contained 5′, 3′, and PolyA structures, the integrity of the transcript could be assessed. To generate full-length non-chimeric (FLNC) reads, the primers, barcodes, and PolyA tails were removed from the sequencing reads and any reads corresponding to intact lanes were excluded. FLNC reads were pooled into full isoforms and hierarchically clustered using minimal AP2 to obtain consensus sequences (unpolished consensus isoforms). Only high-quality isoforms with a prediction accuracy greater than or equal to 0.99 were used for subsequent analyses. The error-correction tool LoRDEC [24] was employed to remove high-quality reads from the RNA sequencing results using a mixed strategy. The BLASTx program (http://www.ncbi.nlm.nih.gov/BLAST/, accessed on 12 October 2022) was used to carry out a BLAST analysis of the isoforms using an E-value of 1 × 10^−5^ against several different databases, such as the Non-Redundant Protein (Nr) database from NCBI (http://www.ncbi.nlm.nih.gov, accessed on 14 October 2022), the Kyoto Encyclopedia of Genes and Genomes (KEGG) database (http://www.expasy.ch/sprot, accessed on 14 October 2022), the Swiss-Prot protein database (http://www.genome.jp/kegg, accessed on 14 October 2022) and the COG/KOG database (http://www.ncbi.nlm.nih.gov/COG, accessed on 14 October 2022). The aim of this analysis was to assess the sequence resemblance between the isoforms and genes from other species. The results of the Nr annotations of isoform were assessed by Blast2GO (version 3.3.5) software [25], where the top 20 subtypes of HSPs with the highest HSP (High-Score Segment Pair) hit rate and no less than 33 occurrences were selected for annotation assessment. WEGO (version 2.0) software was used to classify the functional isoforms [26]. Angle (version 1.0.5) software [27] was then used to obtain the coding sequence (CDS), protein sequence, and non-coding region sequence. The protein-coding sequence of the isoform was compared with the plant TFdb (http://planttfdb.cbi.pku.edu.cn/, accessed on 18 October 2022) using HMMSCAN to predict the transcription factor family.

### 3.4. Analysis of RNA-Seq

FAST [28] was used for further filtering the reads, and the clean high-quality reads were then compared with the three-generation transcriptomic data by RSEM [29] to calculate gene abundance, which was standardized to RPKM (reads per kb per million reads).

Inter-sample repeatability was assessed by calculating Pearson’s correlation coefficient between repeated samples. Therefore, we visualized the correlation heatmap for 30 samples on Omicshare (https://www.omicshare.com/, accessed on 28 February 2023). Principal component analysis (PCA) is a statistical technique that transforms highly correlated variables, and the PCA was performed using the R package. DESeq2 (version 1.22.2) software [30] was used for differential expression of isoforms between two different comparison groups, and the differentially expressed genes were those with a false discovery rate (FDR) < 0.05 and an absolute fold change ≥2, that is, |Log2 (FC)| ≥ 1 genes were differentially expressed genes. Trend analysis can quickly identify the group of genes with the same pattern of expression in responding to the drought stresses in *E. sibiricus*. Therefore, we used all the genes to perform trend analysis on OmicSmart (https://www.omicsmart.com, accessed on 28 February 2023). The relative electrical conductivity (REC) and relative water content (RWC) (Figure S7) from our previous study [22] and all genes from this study were used to perform WGCNA (weighted gene co-expression network analysis) by OmicSmart (https://www.omicsmart.com, accessed 22 March 2023), to identify gene modules associated with phenotypic indicators. In addition, the visualizations of Venn diagrams, volcano map, and heatmaps were built using the free online platform Hiplot (https://hiplot.com.cn/home/index.html, accessed on 17 January 2023).

### 3.5. Quantitative Real-Time PCR (qRT-PCR) Analysis

To provide a further check on the accuracy of the transcriptome data, 11 DEGs from the two genotypes were selected for qRT-PCR validation in the X genotype, and the internal reference gene U2AF was selected based on published research [31]; the qRT-PCR primers are listed in Table S7. Based on the results of fluorescence quantification, the Ct values of these genes were determined, and the representative expression of each gene was then determined using the 2^−△△CT^ method [32]. Each gene has three biological replicates.

## 4. Discussion

As a high-quality forage and ecological restoration grass, *E. sibiricus* is primarily used in alpine regions with arid and semi-arid climates, such as the Qinghai–Tibet Plateau [33]. Therefore, it is essential to evaluate drought resistance and excavate X genes of *E. sibiricus*. At present, the mining of key genes for abiotic stress based on transcriptomics has been realized in many plants [34,35,36,37]. Therefore, transcriptomic studies of different genotypes of *E. sibiricus* under short- and long-term drought stress showed that up-regulating more gene expression in response to more complex and comprehensive pathways is one of the key strategies for drought resistance in *E. sibiricus*. In addition, the MAPK signaling pathway, plant hormone signal transduction pathway, and linoleic acid metabolism pathway play important roles in the drought stress response of *E. sibiricus*.

### 4.1. Phenotypic and Genetic Differences of E. sibiricus in Response to Drought Stress

Based on their phenotypic characteristics, the responses of the two contrasting *E. sibiricus* genotypes to drought stress were significantly different, which could be reflected by the more severe leaf yellowing, wilting, and curling in the W genotype than in the X (Figure S6). Delayed leaf curling in plants has been suggested to be one of the important mechanisms to escape drought by regulating leaf water potential, thus more efficiently absorbing water from the soil [38]. The X genotype exhibited lower REC and higher RWC than the W genotype throughout the drought stress period. Higher RWC and cell membrane stability may help the drought-tolerant genotype to perform various physiological and biochemical processes more efficiently than the drought-sensitive genotype under drought stress, allowing it to tolerate water deficit under drought stress [39,40]. In addition, the REC and RWC of the two genotypes under short- and long-term drought stress showed significant differences compared with the control. The result was consistent with transcriptome sequencing analysis, such as PCA, and hierarchical clustering analysis.

The number of DEGs in the X genotype was higher than that of the W genotype under drought stress. This suggests that the drought-tolerant X genotype has a greater ability to activate and regulate gene expression in response to stress, which may help it cope with drought stress more effectively than the W genotype. In addition, the higher number of DEGs in the X genotype suggests that it experienced less damage at the cellular level, as the up-regulation of genes in response to stress is often associated with the activation of stress-related pathways that can help prevent or repair damage to cellular components caused by stress. The transcription trend observed in soybean (*Aphis glycines*) [41] and upland cotton (*Gossypium hirsutum*) [42] also showed that the drought-tolerant genotype expressed more DEGs than the drought-sensitive genotype under stress conditions. Furthermore, significantly more DEGs were observed in both genotypes only in short-term responses, rather than in long-term drought (Figure S3). This may be because when plants were under stress, they alleviated or adapted to the stress by rapidly regulating the expression of genes [43]. In the early stage of drought, the metabolism mechanism of a plant is not restricted or is less restricted by water shortage, and it can smoothly carry out physiological processes such as transcription and translation [44]. However, in the later stage of drought, the cell membrane of the plant has been damaged by stress, resulting in enhanced permeability of the membrane and strong lipoperoxidation, as well as strong moisture losses. As a result, most of the physiological and biochemical processes cannot function normally due to the water deficit [45]. Consequently, the function of organelles, such as ribosomes and chloroplasts, is also affected, which can lead to a decrease in transcription and translation processes [46,47]. It is therefore important that plants have mechanisms in place to conserve resources and adjust their metabolisms in responding to the stress of drought to maintain cellular function and survival [48].

### 4.2. Metabolic Pathways of E. sibiricus in Response to Drought Stress

By maintaining a higher lipid content under drought stress, plants can resist or adapt to the stress of drought. In the previous research, the lipid content of drought-tolerant cultivars was found to be significantly higher than that of drought-sensitive cultivars [49,50,51]. Linoleic acid is crucial for plant growth, and its oxidation by lipoxygenase produces oxylipoproteins, which are implicated in stimulating the up-regulation of plant defense genes and facilitating plant adaptation to adverse environments [52,53]. In the present study, it was found that most of the genes involved in the degradation of linoleic acid were down-regulated, indicating that the *E. sibiricus* needs to maintain more linoleic acid content to cope with drought stress [54]. Some genes involved in decomposing linoleic acid are not differentially expressed in the early stage of drought, because the plant is experiencing relatively lower drought stress levels. However, in the later stage of drought, to ensure the content of linoleic acid, most of the genes involved in decomposing linoleic acid are down-regulated to reduce linoleic acid breakdown to enhance or adapt to drought stress. Overexpression of the *Arabidopsis thaliana FAD2* gene in tobacco enhances drought tolerance by increasing linoleic acid levels [55]. In addition, linoleic acid can also regulate the water balance of plants, promoting root growth and water uptake, and ultimately enhancing plant drought resistance [56].

ABA is a key signaling factor in responding to drought, regulating plant water status through the regulation of the stomatal conductance and induction of genes responsible for drought resistances [57]. ABA is the main hormone regulating the plant MAPK signaling pathway, which, in turn, triggers the plant antioxidant defense system and inhibits ROS damage under the stress of drought. The MAPK cascade, composed of three types of reversible phosphorylated kinases (MAPKKK, MAPKK, and MAPK), is a fundamental signaling pathway involved in significant biological processes such as plant immunity and hormone responses [58,59]. Plants evolved a response mechanism to sense and transmit stimuli and to activate or inhibit a group of genes responsible for regulating biotic and abiotic stresses during proliferation and development. [60]. The trigger of the MAPK signaling cascade is mainly produced by the activation of reactive oxygen species under stress such as salt, cold, and drought stress [61]. In this study, three types of differentially expressed genes *(MAPKKK17*, *MKK3* and *MPK4*) were detected in the MAPK signaling pathway of *E. sibiricus*. The super-highly differentially expressed *MAPKKK17* gene (*Isoform0027916*) was the most significant gene in drought stress response in *E. sibiricus,* as its expression differentially impacted downstream *MKK3* genes in X and W genotypes. Furthermore, the genes of the *MPK4* module were essential for combating abiotic stresses and contributing to frost resistance [61] and drought tolerance in *Arabidopsis* and cotton [62,63]. In this study, three of the five *MPK4* genes were differentially expressed in the two genotypes, indicating their crucial role in the response of *E. sibiricus* to drought stress. The other two genes were consistently differentially up-regulated in the X genotype, whereas the W genotype had no differential expression after 120 h of drought stress, suggesting that it is one of the important reasons for the difference in drought resistance between the two contrasting genotypes in the MAPK signaling pathway.

Auxins are important regulators of plant growth and development and are also essential in helping plants respond to the stress of drought [64]. Drought stress alters the levels of expression of a variety of auxin-related genes [65], and auxins alter the regulation of many genes, especially the key auxin-responsive genes of the *TIR1*, *Aux/IAA*, *GH3,* and *SAUR* gene families. The auxin receptor *TIR1* represses *Aux/IAA* gene expression through ubiquitination following auxin signaling. In this study, the *TIR1* gene was detected to be up-regulated, and its downstream *Aux/IAA* gene was also up-regulated. This may be because the inhibitory effect of *TIR1* is too small, resulting in a negligible effect on the expression of the *Aux/IAA* gene without any impact on its up-regulated expression. In addition, it was also possible that the ubiquitination process of *TIR1* was interrupted or bound by other substances, resulting in its inability to regulate the downstream *Aux/IAA* genes effectively [66]. Although functional SAUR genes have not been described, *SAUR* proteins have been shown to bind calcium and calmodulin, suggesting a role for calcium ions in the transduction of auxin signals [67]. *GH3* catalyzes the adenylation of specific substrates and is a member of the acyl-adenylate-forming luciferase superfamily [68]. Auxin homeostasis via *GH3* is an essential component of the complex auxin regulatory network involved in stress adaptation in *Arabidopsis* [67]. In this study, differential up-regulation of GH3 and SAUR family members was detected, which may indicate their important roles in the drought resistance of *E. sibiricus*.

### 4.3. Drought-Responsive Transcription Factors of E. sibiricus

During the persistent drought, several TF members, including the WRKY, NAC, MYB, HD-ZIP, ERF, C3H, C2H2, bZIP, MICK, and GRAS families, were found to be continuously up-regulated. ERFs are known to play vital roles in many physiological processes, including plant growth [69], response to biotic/abiotic stresses such as drought, salt, and cold [70], and ethylene and abscisic acid signaling pathways [71]. Previous studies have shown that overexpression of the ERF family member OsDREB2 in rice can improve its drought resistance [72]. Although there has been no research on the drought resistance of the ERF gene in the *E. sibiricus*, we found that there were differentially up-regulated expressions of the ERF gene in two important WGCNA modules, such as *Isoform0011834* (*ERF53*), *Isoform0020075* (*ERF110*), *Isoform0025612* (*DREB2B*), *Isoform0036323* (*ERF4*), *Isoform0026501* (*ERF5*) and *Isoform0028407* (*ERF114*), making them potential candidate genes for improving the drought tolerance of *E. sibiricus*.

WRKY transcription factors are one of the largest families of transcriptional regulators in plants and have a significant role in plant development and various stress responses [73]. Studies have shown that overexpression of wheat WRKY transcription factors in tobacco and *Arabidopsis* can enhance their drought resistance [73,74]. In the present study, 10 WRKY TFs were found to be differentially up-regulated under conditions of stress in two genotypes of *E. sibiricus*, and an important transcription factor, *WRKY24* (*Isoform0012201*), was involved in the MAPK signaling pathway. This transcription factor, together with *WRKY6*, *WRKY9*, *WRKY11*, *WRKY41,* and *WRKY71*, may serve as important WRKY family members involved in the resistance to drought in *E. sibiricus*.

## 5. Conclusions

In this study, we combined the full-length transcriptome with Illumina RNA-seq to explore the molecular mechanism of *E. sibiricus*. The differences in transcriptome and physiological responses between X and W genotypes of *E. sibiricus* under drought stress were compared. A total of 10,659 DEGs were identified, among which the WRKY, NAC, MYB, HD-ZIP, ERF, C3H, C2H2, bZIP, MIKC, Trihelix, GRAS, bZIP, and bHLH were the major TF families associated with the drought resistance of *E. sibiricus*. Furthermore, the MAPK signaling pathway, plant hormone signal transduction, and linoleic acid metabolism may be important pathways in response to drought stress in *E. sibiricus*. This study provides a reference basis for drought-tolerant breeding of *E. sibiricus*.

## Data Availability

All data generated or analyzed during this study are included in this published article and its supplemental data files. The third-generation sequencing and second-generation sequencing raw data of the *E. sibiricus* were deposited in the China National GeneBank DataBase (https://db.cngb.org/, accessed on 6 April 2023) with accession numbers CNP0004264 and CNP0004205.

## Supplementary Materials

The following supporting information can be downloaded at: https://www.mdpi.com/article/10.3390/plants12142719/s1. Figure S1: Selected genes relative expression levels. means of three biological replicates (n = 3) with bars representing SD for each mean; Figure S2: Volcano map of DEGs in four comparison groups of X genotype (A, B, C and D) and W genotype (E, F, G and H); Figure S3: Venn diagram of DEGs in four comparison groups of X genotype (A: up-regulated; B: down-regulated) and W genotype (C: up-regulated; D: down-regulated); Figure S4: Histogram of the number of genes in each module of WGCNA (A); Histogram of the number of TFs in each module of WGCNA (B); Venn diagram of DEGs of two genotypes with genes of darkolivegreen and darkseagreen4 modules (C); Figure S5: The expression heat map of the top 15 hub genes of darkolivegreen and darkseagreen4 modules; Figure S6: Phenotypic morphology of two genotypes of *E. sibiricus* under drought stress; Figure S7: Related physiological indicators of X and W genotypes. (A): Relative water content (RWC). (B): Relative electrical conductivity (REC). Different lower-case letters in the figure indicate significant difference at level of *p* < 0.05. This figure is applied from the research results of Yu et al. (2023) [22], which was the previously published paper by the first author; Table S1: Full-length transcriptome data statistics of three mixed duplicate samples; Table S2: Corrected clustering of full-length non-chimeric sequences; Table S3: Statistics of full-length transcriptome data corrected by transcriptome data; Table S4: Transcriptome data statistics; Table S5: Top 15 key genes information table in the darkolivegreen module; Table S6: Top 15 key genes Information table in the darkseagreen4 module; Table S7: Genes selected for transcriptome verification and their quantitative PCR primer sequences.
