# Supplementary material for: Integrating Full-Length Transcriptome and RNA Sequencing of Siberian Wildrye (Elymus sibiricus) to Reveal Molecular Mechanisms in Response to Drought Stress
Source: Plants (Basel). 2023 Jul 21;12(14):2719. doi: 10.3390/plants12142719 (PMC10385362; doi:10.3390/plants12142719)
Supplement: Supplementary file 1 [file plants-12-02719-s001.zip › Graphical abstracts for figures.pdf]

Figure S1 Selected genes relative expression levels. means of three biological replicates (n=3) with bars representing SD for each mean.

Figure S2 Volcano map of DEGs in four comparison groups of X genotype (A, B, C and D) and W genotype (E, F, G and H).

Figure S3 Venn diagram of DEGs in four comparison groups of X genotype (A: up-regulated; B: down-regulated) and W genotype (C: up-regulated; D: down-regulated).

Figure S4 Histogram of the number of genes in each module of WGCNA (A); Histogram of the number of TFs in each module of WGCNA (B); Venn diagram of DEGs of two genotypes with genes of darkolivegreen and darkseagreen4 modules (C).

Figure S5 The expression heat map of the top 15 hub genes of darkolivegreen and darkseagreen4 modules.

Figure S6 Phenotypic morphology of two genotypes of *E. sibiricus* under drought stress.

Figure S7 Related physiological indicators of X and W genotypes. (A): Relative water content (RWC). (B): Relative electrical conductivity (REC). Different lower-case letters in the figure indicate significant difference at level of $P < 0.05$. This figure is applied from the research results of Yu et al (2023), which was the previously published paper by the first author.
