# Supplementary material for: Integrating Full-Length Transcriptome and RNA Sequencing of Siberian Wildrye (Elymus sibiricus) to Reveal Molecular Mechanisms in Response to Drought Stress
Source: Plants (Basel). 2023 Jul 21;12(14):2719. doi: 10.3390/plants12142719 (PMC10385362; doi:10.3390/plants12142719)
Supplement: Supplementary file 1 [file plants-12-02719-s001.zip › Table S1.pdf]

Table S1 Full-length transcriptome data statistics of three mixed duplicate samples

	Sample	total base (bp)	subreads number	average length	N50
Rawdata	XW-1	11812304736	7071122	1670	1826
	XW-2	27545296228	17756475	1551	1664
	XW-3	8987741778	4582555	1961	2205
Average		16115114247	9803384	1727.33	1898.33
	**Sample**	**Number of reads**	**Number of CCS bases**	**CCS Read Length (mean)**	**Number of Passes (mean)**
Circular consensus sequence (CCS)	XW-1	184176	358439045	1946	26
	XW-2	379004	676694959	1785	31
	XW-3	117403	273472267	2329	27
Average		226861	436202090.3	2020	28
