# Supplementary material for: Integrating Full-Length Transcriptome and RNA Sequencing of Siberian Wildrye (Elymus sibiricus) to Reveal Molecular Mechanisms in Response to Drought Stress
Source: Plants (Basel). 2023 Jul 21;12(14):2719. doi: 10.3390/plants12142719 (PMC10385362; doi:10.3390/plants12142719)
Supplement: Supplementary file 1 [file plants-12-02719-s001.zip › Table S2.pdf]

Table S2 Corrected clustering of full-length non-chimeric sequences

Number of polished high-quality isoforms	Number of polished low-quality isoforms
51215	607
