# Supplementary material for: Integrating Full-Length Transcriptome and RNA Sequencing of Siberian Wildrye (Elymus sibiricus) to Reveal Molecular Mechanisms in Response to Drought Stress
Source: Plants (Basel). 2023 Jul 21;12(14):2719. doi: 10.3390/plants12142719 (PMC10385362; doi:10.3390/plants12142719)
Supplement: Supplementary file 1 [file plants-12-02719-s001.zip › Table S3.pdf]

Table S3 Statistics of full-length transcriptome data corrected by transcriptome data

	Total Number	Total length(bp)	Maximum Length(bp)	Minimum Length(bp)	Average Length(bp)	N50 Length(bp)	GC content
Transcriptome sequencing correction results	51215	95700749	8895	53	1868.61	2039	53.61%
Remove redundant results	40708	76386890	8895	53	1876.46	2058	53.37%
