# Supplementary material for: Integrating Full-Length Transcriptome and RNA Sequencing of Siberian Wildrye (Elymus sibiricus) to Reveal Molecular Mechanisms in Response to Drought Stress
Source: Plants (Basel). 2023 Jul 21;12(14):2719. doi: 10.3390/plants12142719 (PMC10385362; doi:10.3390/plants12142719)
Supplement: Supplementary file 1 [file plants-12-02719-s001.zip › Table S5.pdf]

Table S5 Top 15 key genes information table in the darkolivegreen module

Gene	Symbol	Description	KEGG Pathway	GO Function	GO Process
Isoform0013683	--	unnamed protein product	-	-	-
Isoform0004245	--	unnamed protein product	-	-	-
Isoform0027344	OsI_21320	ferredoxin--NADP reductase, leaf isozyme, chloroplastic-like	Photosynthesis	oxidoreductase activity	sulfur compound metabolic process;sodium ion transport;phenylacetate catabolic process;photosynthesis;cellular metabolic process;oxidation-reduction process
Isoform0013867	--	unnamed protein product	-	-	-
Isoform0010813	--	unnamed protein product	-	-	-
Isoform0011452	NPF3.1	protein NRT1/ PTR FAMILY 3.1-like	-	transmembrane transporter activity	oxoacid metabolic process;nitrogen cycle metabolic process;reactive nitrogen species metabolic process
Isoform0032026	--	hypothetical protein D1007_39638	-	ion channel inhibitor activity	DNA mediated transformation;pathogenesis
Isoform0004074	--	unnamed protein product	-	-	-
Isoform0038345	OsI_21320	ferredoxin--NADP reductase, leaf isozyme, chloroplastic-like	Photosynthesis	oxidoreductase activity	sodium ion transport;phenylacetate catabolic process;oxidation-reduction process
Isoform0031958	--	unnamed protein product	-	-	-

Isoform0035778	At4g27700	rhodanese-like domain-containing protein 14, chloroplastic	-	-	selenium compound metabolic process;tRNA modification
Isoform0014603	CRK20	putative cysteine-rich receptor-like protein kinase 20 isoform X2	-	protein kinase activity;ATP binding	protein phosphorylation
Isoform0036135	Cht4	chitinase 5-like	Amino sugar and nucleotide sugar metabolism;	chitinase activity;chitin binding	chitin catabolic process;cell wall macromolecule catabolic process
Isoform0024060	ZB8	phenylalanine ammonia-lyase-like	Biosynthesis of secondary metabolites;Phenylalanine metabolism	histidine ammonia-lyase activity	histidine catabolic process;biosynthetic process;phenylpropanoid metabolic process
Isoform0005672	Os03g0733400	unnamed protein product	-	protein dimerization activity	-
