# Supplementary material for: Integrating Full-Length Transcriptome and RNA Sequencing of Siberian Wildrye (Elymus sibiricus) to Reveal Molecular Mechanisms in Response to Drought Stress
Source: Plants (Basel). 2023 Jul 21;12(14):2719. doi: 10.3390/plants12142719 (PMC10385362; doi:10.3390/plants12142719)
Supplement: Supplementary file 1 [file plants-12-02719-s001.zip › Table S6.pdf]

Table S6 Top 15 key genes Information table in the darkseagreen4 module

Gene	Symbol	Description	KEGG Pathway	GO Function	GO Process
Isoform0032397	At1g32060	phosphoribulokinase, chloroplastic	Carbon metabolism;Carbon fixation in photosynthetic organisms	pantothenate kinase activity;uridine kinase activity;ATP binding;kinase activity	coenzyme A biosynthetic process;uridine metabolic process
Isoform0023682	CSP41B	chloroplast stem-loop binding protein of 41 kDa b, chloroplastic	-	oxidoreductase activity, transferase activity, transferring glycosyl groups	carbohydrate metabolic process
Isoform0025330	CSP41B	chloroplast stem-loop binding protein of 41 kDa b, chloroplastic	-	oxidoreductase activity, transferase activity, transferring glycosyl groups	carbohydrate metabolic process;galactose metabolic process
Isoform0026146	CSP41B	chloroplast stem-loop binding protein of 41 kDa b, chloroplastic	-	oxidoreductase activity, transferase activity, transferring glycosyl groups	carbohydrate metabolic process;galactose metabolic process
Isoform0038641	TRXM	unnamed protein product	-	antioxidant activity;oxidoreductase activity	cell redox homeostasis； cellular response to toxic substance； response to stress； reactive oxygen species metabolic process
Isoform0038372	TRXM	unnamed protein product	-	antioxidant activity;oxidoreductase activity	cell redox homeostasis； cellular response to toxic substance； response to stress； reactive oxygen species metabolic process

Isoform0022542	At3g55800	sedoheptulose-1,7-bisphosphatase, chloroplast precursor	Carbon metabolism;Carbon fixation in photosynthetic organisms	hydrolase activity；carbohydrate phosphatase activity; phosphoric ester hydrolase activity	polysaccharide metabolic process；glucan biosynthetic process；reductive pentose-phosphate cycle
Isoform0023148	At3g55800	sedoheptulose-1,7-bisphosphatase, chloroplast precursor	Carbon metabolism;Carbon fixation in photosynthetic organisms	hydrolase activity；carbohydrate phosphatase activity; phosphoric ester hydrolase activity	polysaccharide metabolic process；glucan biosynthetic process；reductive pentose-phosphate cycle
Isoform0023621	RPS1	unnamed protein product	Ribosome	DNA-directed RNA polymerase activity；polyribonucleotide nucleotidyltransferase activity	RNA catabolic process;mRNA catabolic process;translation
Isoform0027271	EO	2-methylene-furan-3-one reductase	-	oxidoreductase activity；S-(hydroxymethyl)glutathione dehydrogenase activity	threonine catabolic process；oxidation-reduction process
Isoform0016693	RPS1	unnamed protein product	Ribosome	DNA-directed RNA polymerase activity；polyribonucleotide nucleotidyltransferase activity	mRNA catabolic process;translation
Isoform0026503	At3g55800	sedoheptulose-1,7-bisphosphatase, chloroplast precursor	Carbon metabolism;Carbon fixation in photosynthetic organisms	hydrolase activity; carbohydrate phosphatase activity; phosphoric ester hydrolase activity	polysaccharide metabolic process；glucan biosynthetic process；reductive pentose-phosphate cycle
Isoform0038101	GDCSH	Glycine cleavage system H protein, mitochondrial	Biosynthesis of secondary metabolites;Carbon	catalytic activity;dihydrolipoyllysine-residue acetyltransferase activity	organic acid metabolic process；ellular amino acid metabolic process

			metabolism;Glyoxylate and dicarboxylate metabolism;Glycine, serine and threonine metabolism		
Isoform0020571	RPS1	unnamed protein product	Ribosome	DNA-directed RNA polymerase activity；polyribonucleotide nucleotidyltransferase activity	RNA catabolic process;mRNA catabolic process;translation
Isoform0028235	CSP41B	chloroplast stem-loop binding protein of 41 kDa b, chloroplastic	-	dTDP-4-dehydrorhamnose reductase activity；dTDP-4-dehydrorhamnose reductase activity	carbohydrate metabolic process;galactose metabolic process;O antigen biosynthetic process;oxidation-reduction process
