# Supplementary material for: Integrating Full-Length Transcriptome and RNA Sequencing of Siberian Wildrye (Elymus sibiricus) to Reveal Molecular Mechanisms in Response to Drought Stress
Source: Plants (Basel). 2023 Jul 21;12(14):2719. doi: 10.3390/plants12142719 (PMC10385362; doi:10.3390/plants12142719)
Supplement: Supplementary file 1 [file plants-12-02719-s001.zip › Table S7.pdf]

Table S7 Genes selected for transcriptome verification and their quantitative PCR primer sequences

Gene ID	F	R
Isoform0015152	TACTCAACAAGGAGGTGTGATG	CTCTACCATGTGCTTGTCTTCT
Isoform0035368	GAAAGCTTCGAGGGTACCAAA	GGAGCTGTTGCTGCTGATAA
Isoform0021975	TCTCCAAGCTCTACGTCCA	CGCTGCACCTTCTTCTTCA
Isoform0022145	AGGAGCTCATCCTCCACTAC	GACACCGTTGGGTACTTACG
Isoform0025450	TCTGCAGGCGATGTCAAG	CTTTCGCTTCCCGGTGAT
Isoform0016436	CGCGCAAGTACCACTACTC	GTGTGGGCGAAGAAGCA
Isoform0023226	GGTACAAGACGAAGATCCTAGC	CACCGGCGTCTTCTGTAATA
Isoform0026062	CGCCTGATGTCACTGCT	AGCATCTGCGGCATCTC
Isoform0026049	AGCATTGGAGAAGGGAAGATG	TCCTTCACGATGATCTGGTTG
Isoform0025505	TGCTCTACCCAGAGGAGTTT	CTCATCCTTGTACTCTGCCATC
Isoform0003383	GTTTGATGGCTCTTCGCTTTC	CTCCAAGACTGTGCCAATAGT
U2AF	ATCGCTGCTCTCGCATCCATAAC	TGCTGCTGCCTGATCTTCCTCT
