# Supplementary figures and images for: Integrating Full-Length Transcriptome and RNA Sequencing of Siberian Wildrye (Elymus sibiricus) to Reveal Molecular Mechanisms in Response to Drought Stress
Source: Plants (Basel). 2023 Jul 21;12(14):2719. doi: 10.3390/plants12142719 (PMC10385362; doi:10.3390/plants12142719)

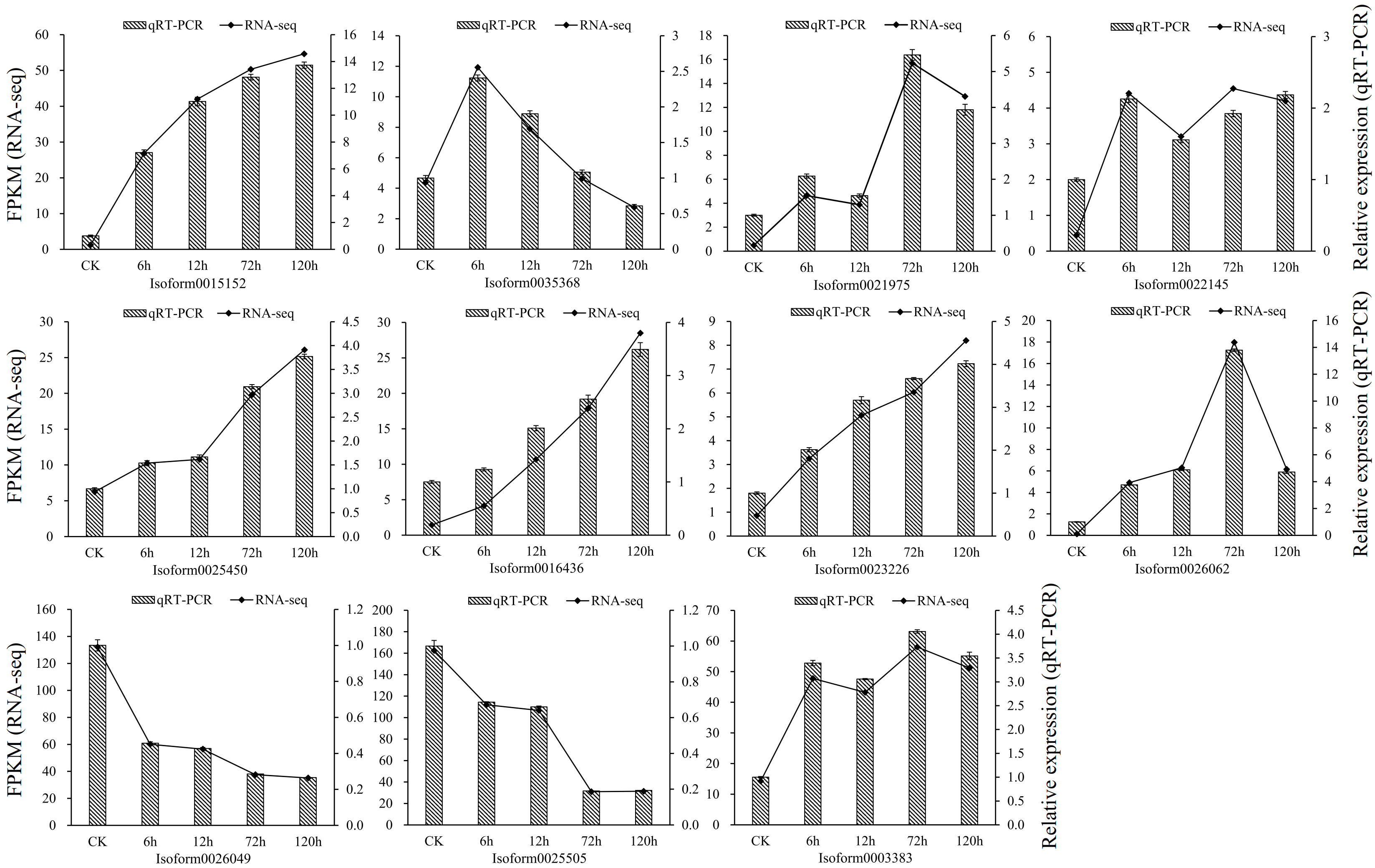

Supplement: Supplementary file 1 [file plants-12-02719-s001.zip › Figure S1.tif]

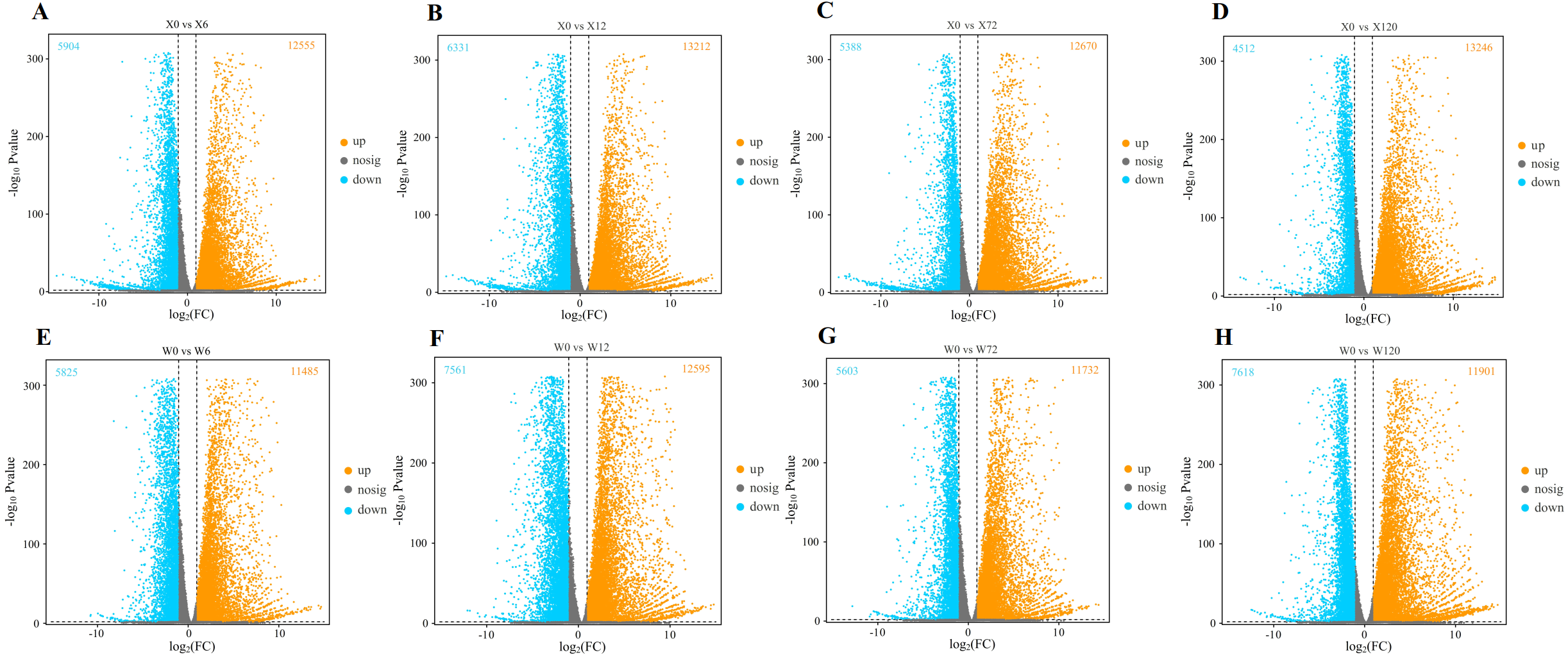

Supplement: Supplementary file 1 [file plants-12-02719-s001.zip › Figure S2.tif]

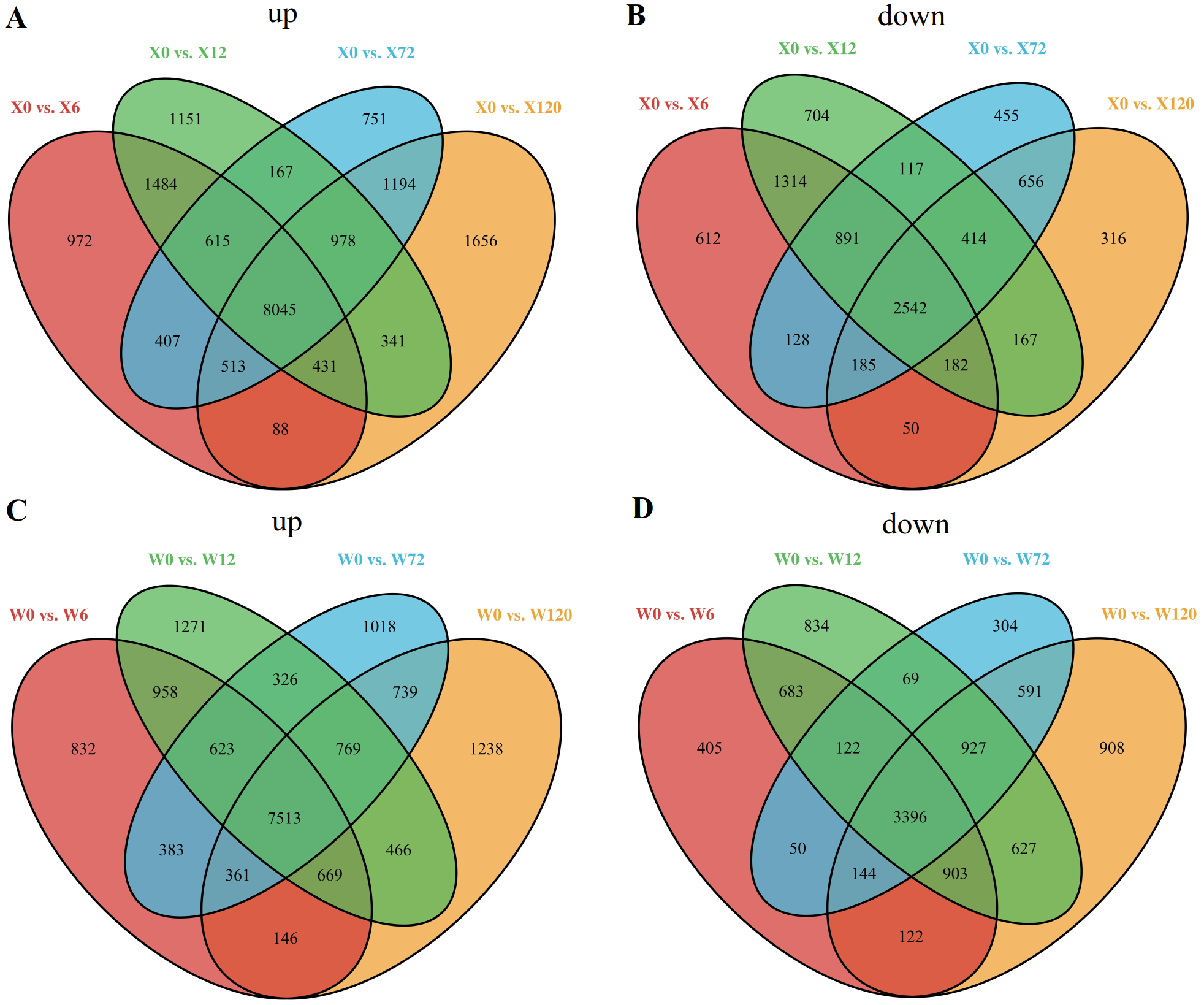

Supplement: Supplementary file 1 [file plants-12-02719-s001.zip › Figure S3.tif]

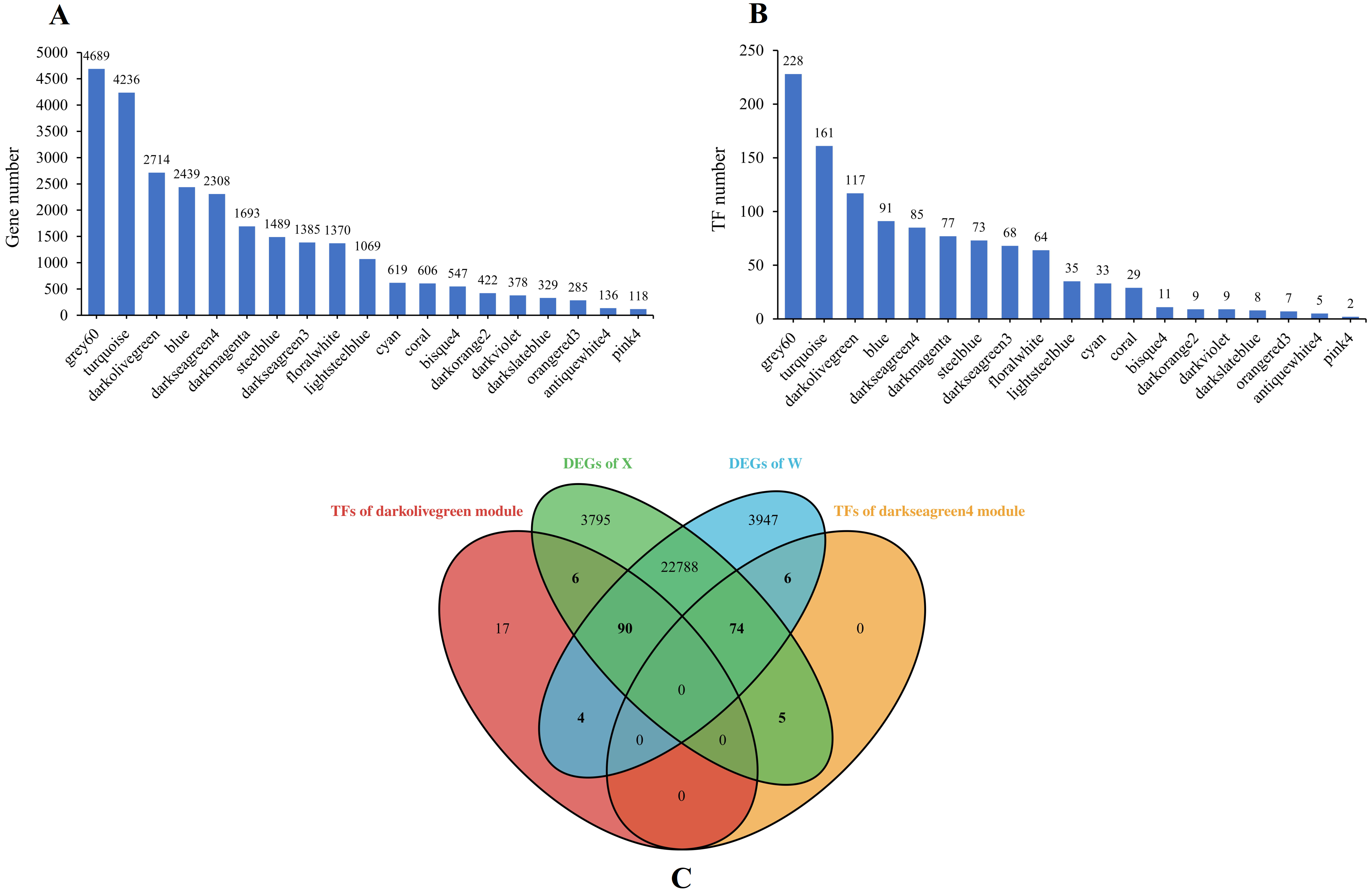

Supplement: Supplementary file 1 [file plants-12-02719-s001.zip › Figure S4.tif]

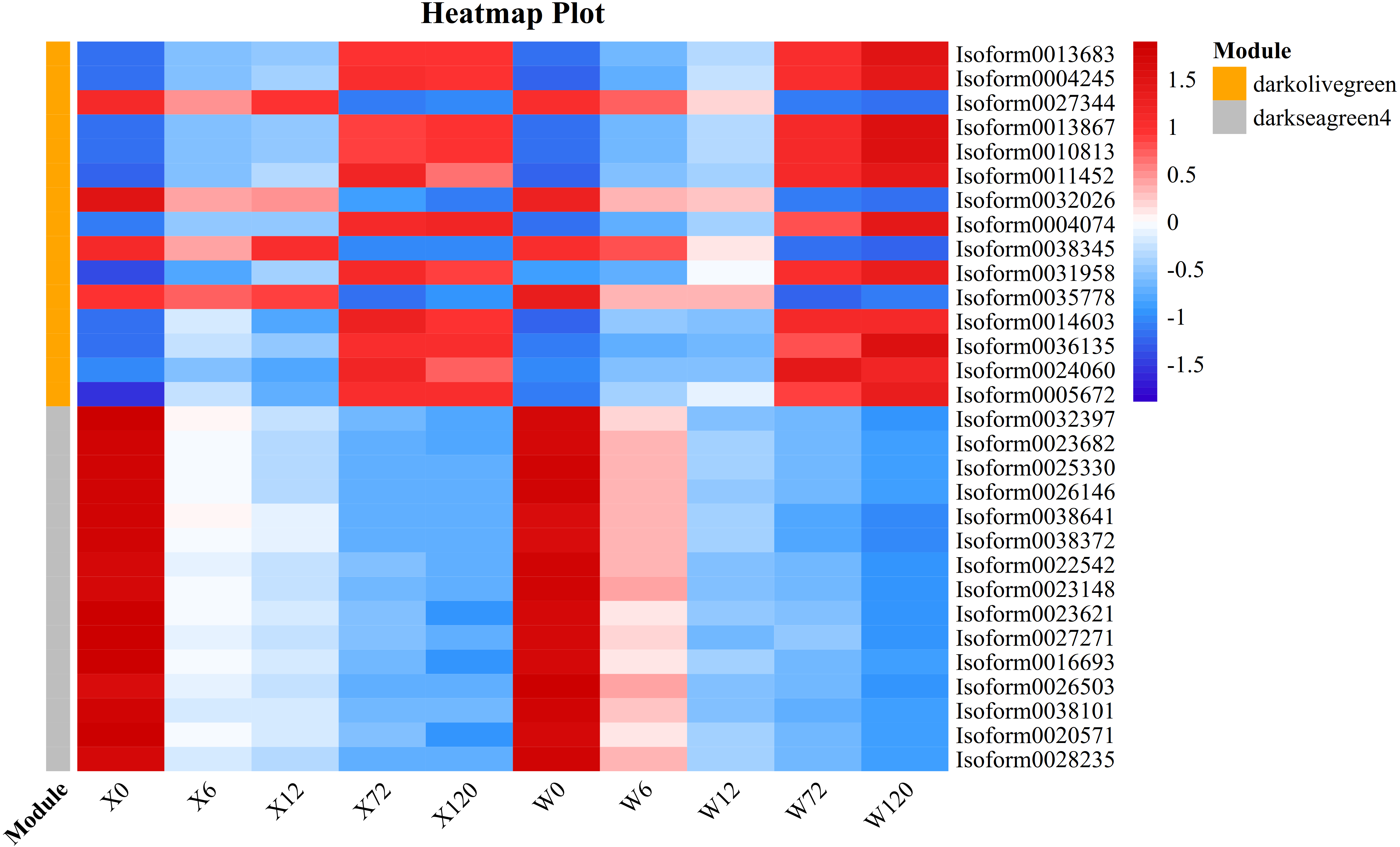

Supplement: Supplementary file 1 [file plants-12-02719-s001.zip › Figure S5.tif]

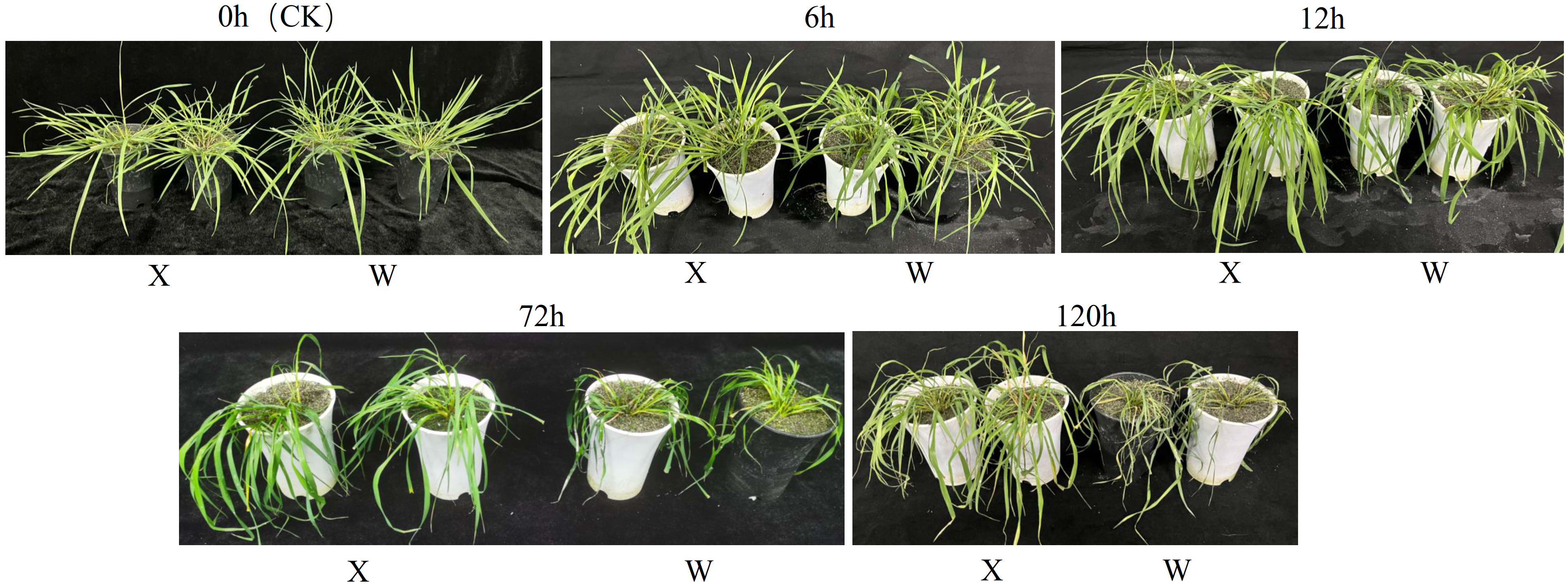

Supplement: Supplementary file 1 [file plants-12-02719-s001.zip › Figure S6.tif]

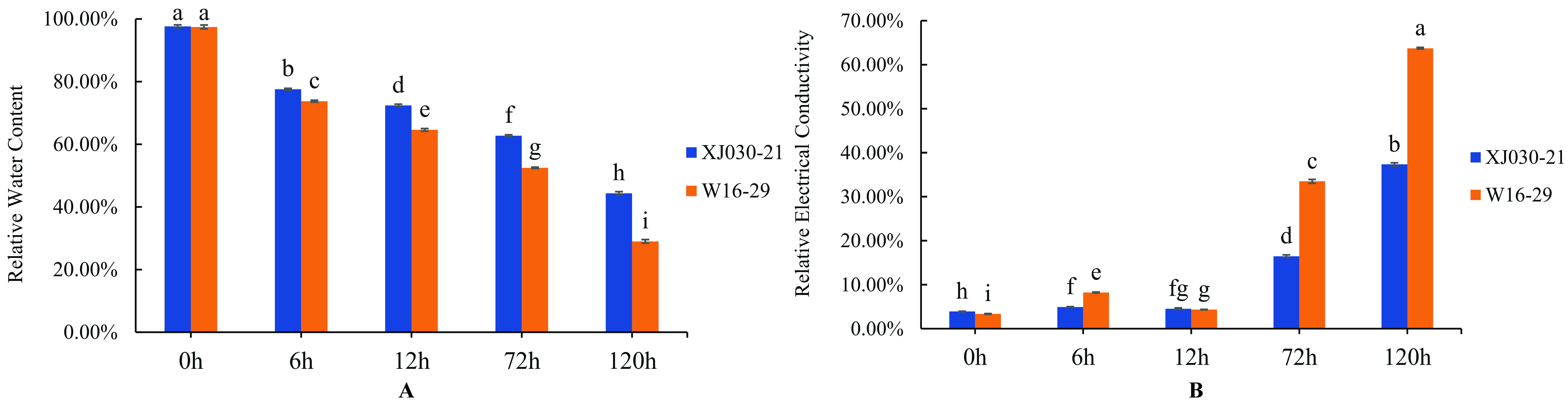

Supplement: Supplementary file 1 [file plants-12-02719-s001.zip › Figure S7.tif]
